# When the water stops flowing in humanitarian emergencies

**DOI:** 10.2471/BLT.22.020722

**Published:** 2022-07-01

## Abstract

Increasingly frequent humanitarian emergencies are highlighting the need for more effective collaboration on water, sanitation and hygiene response. Lynn Eaton reports.

Omar El Hattab will never forget the night the children were killed in Aleppo. He was in the Syrian city at the time, leading the United Nations Children’s Fund (UNICEF) response to a humanitarian crisis that had flared up when groups opposing the government had deliberately cut off the water supply to part of the city, leaving 1.5 million people without running water.

“It happened on the night of August 14th, 2015,” El Hattab remembers. “The children were filling jerry cans and plastic bottles from a water point that we had set up in Sa’a Square in the west of the city. A mortar shell came in from eastern Aleppo and killed 12 of them. I cried when I heard the news.”

Now serving as UNICEF’s Senior Advisor for Water, Sanitation and Hygiene (WASH), El Hattab is watching the humanitarian crisis unfold in Ukraine, where, once again, water supplies are being impacted by intensive shelling and missile strikes on Ukraine’s cities and infrastructure.

According to the United Nations Office for the Coordination of Humanitarian Affairs, an estimated 13 million people are currently in need of WASH assistance in Ukraine, some three million of them children.

While the conflict in Ukraine is grabbing headlines, El Hattab is quick to point out that it is not the only active war zone, nor the only conflict where water infrastructure and supply are being impacted.

“Water has been affected in almost every conflict-related emergency to which UNICEF has responded in the past few years,” he says, citing examples in Ethiopia, Iraq, the Syrian Arab Republic and Yemen. UNICEF launched a Water Under Fire campaign which included the publication of a three-volume report to draw attention to the issue in March 2019.

Climate change is also driving a rise in the number of WASH emergencies, increasing the severity and incidence of droughts, flooding and storms. Recent examples include the severe flooding experienced in the Zambezia, Nampula and Tete provinces of Mozambique when the country was hit by storm Ana in January of 2022.

The low-lying coastal district of Maganja da Costa was one of the areas affected. “When Storm Ana struck, 2511 latrines and 20 boreholes were destroyed,” says Charmaine Consul Goncalves, Mozambique head of programmes for WaterAid, an international nongovernmental organization, focused on water, sanitation and hygiene. WaterAid has since been supporting a local organization, Kukumbi, to provide safe water and sanitation in the area.

As Goncalves points out, the damage to the district’s WASH capacity had significant knock-on effects, including damage to local health facilities. At the Mugeba Health Centre in Tapua, Zambezia, two water sources were flooded and damaged as were the latrines. “Staff had to provide medical care to 400 patients without clean water or sanitation,” Goncalves says.

“Water has been affected in almost every [recent] conflict-related emergency.”

Omar El Hattab

This kind of domino effect is typical in WASH emergencies. “When the flow of water stops or water sources are contaminated, a lot of activities shut down, including the provision of health care,” says El Hattab, pointing out that it is in these circumstances that diarrhoeal diseases, including cholera, can spread.

Emergency response typically begins with one focus: the delivery of safe drinking-water or the chemicals needed to make water safe. UNICEF’s response to the attack on Ukraine began in this way, and as of 17 May, the organization had ensured access to safe water for nearly 2.1 million people, including through water trucking, bottled water distribution and supporting water availability in collective centres. In addition, UNICEF had reached over 141 500 people with WASH supplies.

But, as El Hattab explains, water trucking is only a stop-gap. Addressing WASH emergencies requires longer term interventions and these quickly become complex, requiring action on many fronts, notable among which, infrastructure repair.

“In an important sense the water problem is an infrastructure problem,” says El Hattab, pointing to Ukraine’s cities as an example, where relentless shelling and missile strikes have devastated pumping and drainage infrastructure, including the power utilities needed to run it. “Without power you can’t pump or drain,” El Hattab explains. “Without power you can’t treat wastewater.”

Working in collaboration with WASH partners, UNICEF is providing equipment and parts needed to repair damaged water systems in the Ukrainian cities of Sumy and Chernihiv and, as of late May, had supplied 11 tonnes of liquified chlorine gas to Kharkiv.

Fixing infrastructure is not just complex, it requires foresight and planning. “You don’t just install infrastructure in the way you might install portacabins,” says Emmett Kearney, Senior WASH officer at the United Nations High Commission for Refugees (UNHCR). “You need to think longer term, work out what all the needs are, who has funding for what, and who can fill the gaps. You also have to prioritize within each sector and draw up a plan in consultation with the local authorities, refugees and other stakeholders. And that all has to happen early on because the decisions made in the first weeks and months of an emergency have an impact on the type of services available in five or ten years.”

Margaret Montgomery, a WASH expert at the World Health Organization (WHO), agrees, emphasizing the need for collaboration in effective emergency response that includes forward planning.

“The truth is we can achieve so much more by coming together in terms of technical expertise, financial resources, general dynamics and energy,” she says.

It was to encourage such collaboration that, in 2005, humanitarian organizations adopted the cluster approach, to coordinate humanitarian action when a national government requests international support.

Comprised of organizations working in 11 humanitarian sectors, including WASH response, clusters can include nongovernmental organizations, United Nations agencies, the Red Cross/Red Crescent Movement, and government bodies. The Global WASH Cluster (GWC) is led by UNICEF.

Getting all those participants to come together in what are often high-pressure situations is challenging although examples of collaboration abound, and not just among the biggest agencies. In Mozambique, for example, WaterAid works with partners in the WASH cluster to agree the overall goals following a disaster and decide what each of them can achieve.

But collaboration is not always easy. UNHCR’s Kearney lists challenges that include the differing missions and agendas of the various organizations involved.

“The decisions made in the first weeks impact the type of services that will be available in five years.”

Emmett Kearney

Bruce Gordon, Geneva-based unit head for WASH at WHO, notes the technical and institutional challenges arising from interagency, intersectoral collaboration. “Typically, WHO gets engaged if we see a threat of cholera-related or other waterborne diseases emerging. Then, what normally happens is someone says: ‘Have you talked to the WASH people?’, meaning someone in another agency such as UNICEF. It can get quite complex, which is why we are trying to do more together.”

UNICEF’s El Hattab notes that there is also scope for greater local engagement in WASH response, notwithstanding the many examples where such engagement is a reality. In Ukraine, for example, UNICEF leads meetings with other WASH agencies and government authorities to agree on priorities that include integrating services for children, supporting critical services to prevent the collapse of the water supply, and holding weekly WASH meetings to assess the repairs needed to the water supply and monitor water quality.

How best to encourage local governmental and/or nongovernmental agencies to get involved was a central topic of discussion at the first World Humanitarian Summit which took place in Istanbul in May 2016. To support local engagement, GWC mobilized a Field Support Team which is tasked with helping National Humanitarian WASH Coordination Platforms, which, as the name suggests, are intended to consolidate local emergency response capacity and action.

National platforms are widely considered to be a good idea, but not everyone is convinced that they have the resources they need. “In many countries the national WASH coordination platforms continue to be under-funded,” says El Hattab, voicing a commonly expressed concern.

Montgomery believes that one way to encourage more local engagement and buy-in would be for lead agencies to make less use of visible identification or branding. “The problem with slapping a logo on a pump or water tank is that the local community automatically sees it as the agency’s property or project from the start, undermining any hopes of local ownership, sustained operation, or building back better,” she says.

Kearney sees other benefits to setting aside the trappings of institutional identity, and the thinking that can go with it. “It’s really important for actors to think first about the needs of affected populations, not their institutional priorities, to collectively agree on what actions to take and who does what,” he says. “There is plenty for everyone to do and no prospect of that changing in the foreseeable future.”

**Figure:**
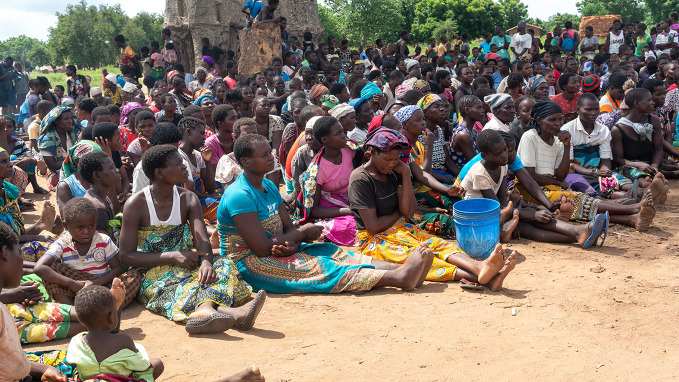
Community members learn about WASH hazards after Storm Ana in Tapua, Zambezia in Mozambique.

WaterAid/Francis Chipanda

**Figure:**
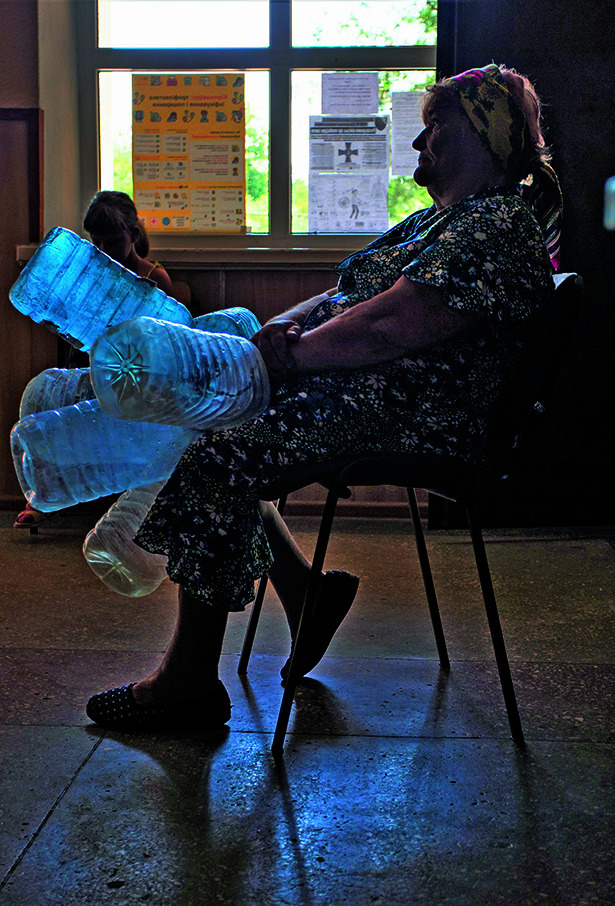
An elderly woman waits to fill bottles with water in Pavlopil, Ukraine.

UNICEF/Aleksey Filippov

